# From quiescence to hypertrophy: existing and emerging genetic targets to fine-tune myogenesis in cultivated muscle

**DOI:** 10.3389/fbioe.2026.1949264

**Published:** 2026-09-25

**Authors:** Lucy Wilkinson, David Tosh, Marianne Ellis

**Affiliations:** 1 Department of Chemical Engineering, The University of Bath, Bath, United Kingdom; 2 Department of Life Sciences, The University of Bath, Bath, United Kingdom

**Keywords:** cell line engineering, cell signalling, cultivated meat, muscle, myogenesis, satellite cells

## Abstract

Cultivated meat (CM) offers a promising alternative to conventional meat with the potential to address the sustainability, animal welfare, and public health concerns associated with intensive animal agriculture. The scalability, economic viability and quality of a CM product are likely to be reliant on high-performing muscle cell lines capable of rapid proliferation, efficient differentiation and a high protein yield relative to culture volume. Although early research has prioritised the expansion of undifferentiated cell biomass, differentiated muscle cells will likely be important for replicating the structural and nutritional properties of conventional meat. Cell line engineering offers a powerful method to achieve this by enabling precise control over cellular functions while reducing the cost and complexity of a bioprocess. To date, cell line engineering has been deployed in the context of CM to improve cell characteristics, such as immortality, proliferation rate, or metabolic efficiency. However, many of the complex signalling pathways that regulate myogenesis and skeletal muscle hypertrophy remain underexplored in agriculturally-relevant cell lines. This review explores the key existing and emerging genetic targets for enhancing myogenesis in the context of CM, including TGF-β, MEK/ERK, PI3K/AKT/mTOR and Wnt signalling pathways. Engineering these pathways has the potential to fine-tune the balance between proliferation and differentiation or induce hypertrophy and increase protein yield without the need for costly media additives. The integration of advanced genome editing technologies with rigorous quality control and regulatory systems could significantly improve the performance of muscle-derived cells for scalable CM production.

## 1 Introduction

By 2050, meat consumption is expected to have risen by approximately 50% (Sievert, 2023). Meeting this demand would require increased intensity of animal agriculture, an industry that already contributes ∼16% of anthropogenic greenhouse gas emissions (Okamoto et al., 2020; Twine, 2021; Chen et al., 2024). Further, the rise of antimicrobial resistance and outbreaks of zoonotic diseases, such as swine flu or COVID-19, continue to threaten the resilience of the global food systems providing animal protein (Kelbrick et al., 2023). Against this backdrop of environmental and public health issues associated with conventional animal agriculture, cultivated meat (CM) has emerged as an alternative with the potential to offer a healthier, ethical, and more sustainable source of dietary protein compared to conventional meat (Huang et al., 2024).

CM technology combines tissue engineering principles and large-scale cell culture to produce animal muscle and fat cells as food ingredients, with most CM bioprocesses aiming to produce skeletal muscle from the differentiation of stem cells, such as muscle satellite cells (MuSCs), induced pluripotent stem cells (iPSCs) or embryonic stem cells (ESCs) (Olenic et al., 2024). Developing scalable CM bioprocesses that will allow for cost-competitive products is undoubtedly a challenge arising from process complexities, high costs and the shortcomings of mammalian cells for industrial scale culture (Riquelme-Guzmán et al., 2024; Guan et al., 2025). Cell line engineering represents a powerful tool to improve cell performance and reduce costs of a CM bioprocess. To date, cell line engineering has been employed in the context of CM to immortalise cell lines or reduce reliance on expensive growth factors such as fibroblast growth factor-2 (FGF-2) (Krieger et al., 2022; Stout et al., 2024). While initial cell line development will cost time and money, an engineered cell line can permanently reduce or eliminate dependence on exogenous growth factors or other additives, making this method of bioprocess optimisation relatively low cost.

Although cell line engineering has been successfully deployed in the context of CM to improve cell performance, the lack of complete differentiation in many myogenic cultures remains a shortcoming, with fusion indices of around 50% commonly observed (Melzener et al., 2024). Complete differentiation will likely be important for obtaining comparable nutritional, sensory and market value qualities to conventional meat (Melzener et al., 2024). During myogenesis, Pax7(+) MuSCs that reside in adult skeletal muscle are activated and give rise to proliferating myoblasts. Myoblasts eventually undergo differentiation, fusing to form multinucleated myotubes which themselves mature into myofibres. In adult skeletal muscle under stress or injury, myofibres may undergo hypertrophy and further grow in diameter. Each of these cell phenotypes is delineated by the expression of key transcription factors, known as myogenic regulatory factors (MRFs), namely Myf5, MyoD, myogenin and MRF4 (Figure 1).

**FIGURE 1:**
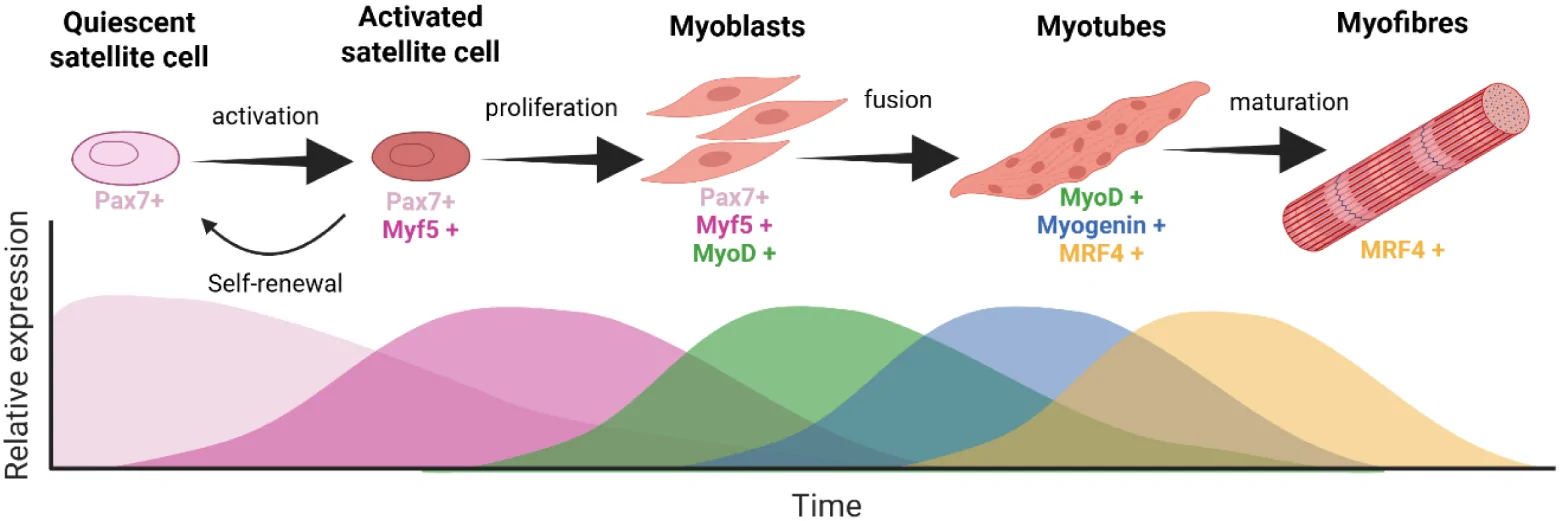
Key transcription factor expression during myogenesis. Activation and differentiation of muscle satellite cells (MuSCs) is controlled by a network of transcription factors, including Pax7 and the myogenic regulatory factors (MRFs). Pax7 is a canonical MuSC marker, which wanes upon MuSC activation. Early MRFs include myogenic factor 5 (Myf5) and myoblast determination protein 1 (MyoD). Later in differentiation, MRF4 and myogenin expression promote terminal differentiation and fusion into myotubes.

Many cell signalling pathways are involved in regulating myogenesis, including the TGF-β signalling pathway (Von Maltzahn et al., 2012), JAK/STAT signalling pathway (Moresi et al., 2019), Wnt signalling (Rochat et al., 2004; Abu-Elmagd et al., 2010; Han et al., 2011; Von Maltzahn et al., 2012) and the PI3K/AKT signalling pathway (Yeh et al., 2014; Guan et al., 2022). Recently, the potential for modulating these pathways by external biochemical and environmental cues to improve myogenesis and establish mature muscle architecture has been explored (Melzener et al., 2024; Jung et al., 2025). How these pathways can be modified through cell line engineering–including gene editing (insertion of cisgenes or specific base edits into the host cell’s genome) or genetic modification (insertion of transgenes) – remains an exciting question for the CM field.

With a focus on fine-tuning myogenesis to produce skeletal muscle as a protein ingredient in CM products, this review aims to provide insights into the existing cell line engineering strategies as well as emerging genetic targets so far not investigated in the context of CM. We also discuss the potential limitations of cell line engineering as a strategy to improve cell line performance, considering regulatory hurdles, consumer acceptance and cell line stability. The findings are intended to inform future research and development of cell lines for enhanced protein production in cultivated muscle cells while reducing bioprocessing cost and complexity.

## 2 Increasing proliferation capacity by controlling cell fate

Maximizing cell proliferation prior to differentiation is essential for achieving high biomass yields in a cultivated meat bioprocess. MuSCs possess the intrinsic ability to both self-renew and differentiate, but their natural tendency to exit the cell cycle and commit to myogenesis limits their expansion potential *in vitro* (Melzener et al., 2024). Controlling the balance between proliferation and differentiation through targeted genetic interventions represents a potential strategy to improve process efficiency. This section highlights potential signalling pathways and genetic targets that can be leveraged to extend the proliferative phase of MuSCs, while preserving their differentiation capacity.

### 2.1 Existing strategies to enhance proliferation through cell cycle modification

Primary cells that are not biologically immortal exhibit replicative senescence, primarily due to telomere shortening (Soice and Johnston, 2021; Stout et al., 2023). The development of cell lines overexpressing telomerase reverse transcriptase (TERT) allows this replicative limit to be surpassed. Other immortalisation strategies include targeting key factors involved in cell cycle regulation such as the simultaneous inactivation of the tumour suppressor genes p53 and Rb (Nachtigal et al., 1989; Delia et al., 2003; Guo et al., 2022) or overexpression of the cell cycle regulator cyclin-dependent kinase 4 (CDK4) to bypass senescence (Shiomi et al., 2011; Stout et al., 2023).

Immortalised cell lines often exhibit lower doubling times than their primary cell counterparts due to a reduced reliance on growth factors, weakened cell cycle checkpoints and a reprogramming towards continuous proliferation (Riquelme-Guzmán et al., 2024). Therefore, immortalised cell lines are generally considered a requirement to enable generation of large quantities of edible tissue and overcome the limitations of primary cells, including replicative senescence, donor-to-donor variability and the need for repeat biopsies (Riquelme-Guzmán et al., 2024).

### 2.2 TGF-β/Smad and notch signalling delay differentiation

The expression of MRFs can be inhibited by exogenous factors, maintaining cells in an undifferentiated state and promoting MuSC expansion. One such group of factors is the bone morphogenic proteins (BMPs), belonging to the transforming growth factor-β (TGF-β) superfamily. There are three main components of the canonical TGF-β signal transduction pathway: the ligand, the receptors and the intracellular mediators (Smads). For both TGF-β and BMP subfamilies, signal transduction is initiated through the binding of a ligand to its type II receptor which forms a heteromeric complex with its corresponding type I receptor. The activated receptor complex subsequently phosphorylates a receptor-regulated Smad protein that then interacts with a co-Smad and is translocated into the nucleus where it regulates transcription through interaction with Smad binding partners.

Canonical TGF-β signalling activates Smad2 and Smad3 which oligomerise and translocate into the nucleus and block the binding of MyoD to E-box elements of skeletal muscle-specific genes (Figure 2A) (Jen et al., 1992; Katagiri et al., 2002; Korchynskyi and Ten Dijke, 2002). This manifests as delayed fusion and diminished myotube size and reduced MRF expression, but these effects can be relieved by inhibition of TGF-β signalling (Jung et al., 2025). The BMP subfamily regulates target gene expression via phosphorylation of Smad1/5/8 and is negatively regulated by secreted BMP inhibitors such as Noggin. The most common BMP targets are Id genes (Inhibitor of differentiation/DNA-binding proteins), which form inactive complexes with basic helix-loop-helix (bHLH) muscle regulatory factors such as MyoD, thereby inhibiting muscle-specific gene activation. The osteogenic effect of BMPs has been intensively studied in C2C12s, which will differentiate into osteoblasts upon BMP stimulation. Because of this observation, BMP signalling has not been extensively studied in the context of adult myogenesis, however recent work has shown that BMPs retain C2C12s in a proliferative state and inhibition of BMP signalling results in cell cycle exit and myotube formation (Friedrichs et al., 2011).

**FIGURE 2:**
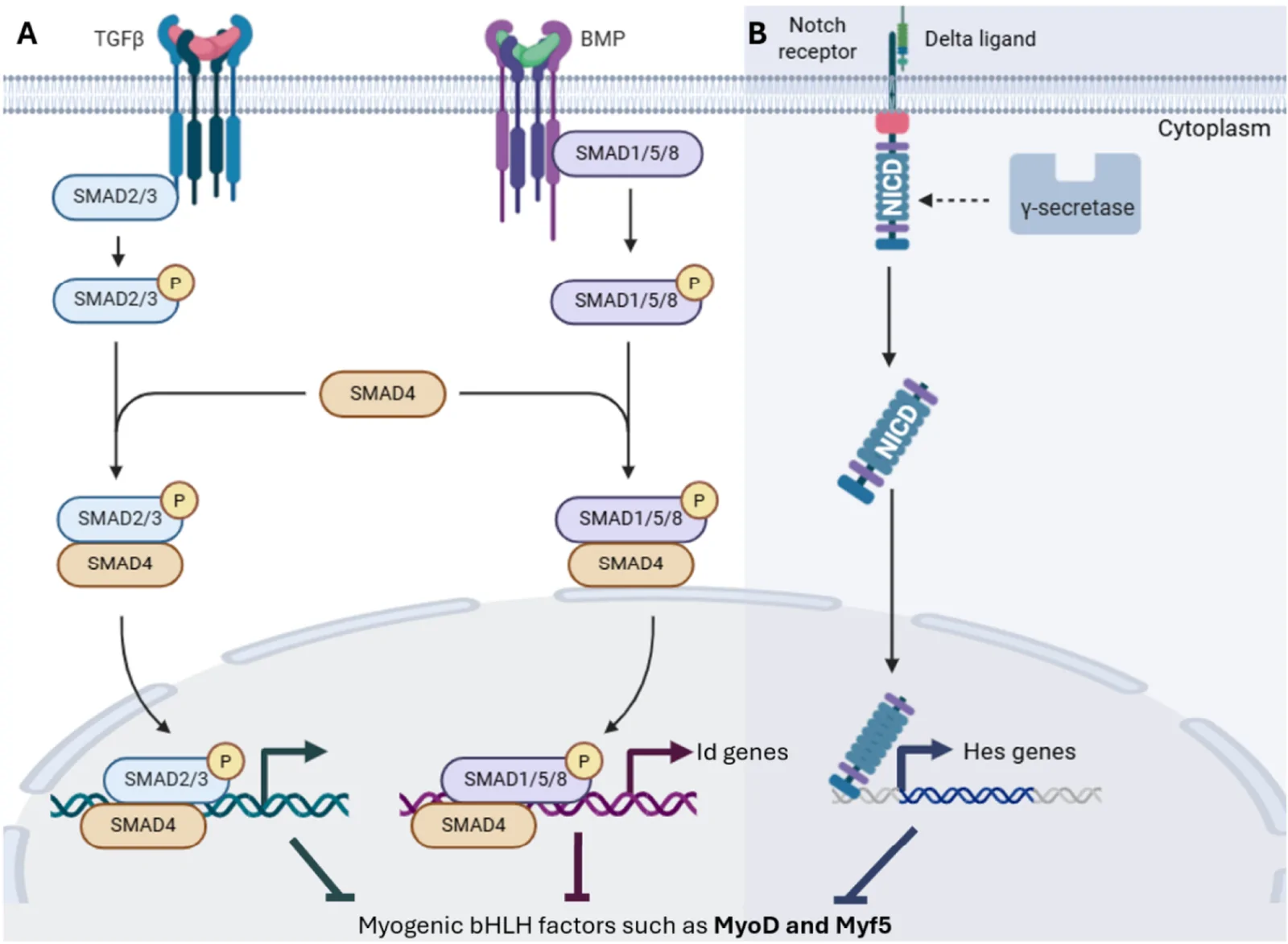
The negative regulation of myogenic differentiation by TGF-β signalling and Notch signalling. **(A)** Binding of TGF-β or BMP ligands to their respective type II receptors promotes formation and activation of a heteromeric receptor complex with type I receptors. The constitutively active kinase domain of the type II receptor phosphorylates the type I receptor, which subsequently phosphorylates receptor-regulated Smad proteins. Phosphorylated Smads form complexes with co-Smads and translocate to the nucleus, where they regulate transcription of target genes that inhibit myogenic differentiation. **(B)** Notch receptor activation induces intracellular signalling that promotes expression of Hes genes. Hes proteins inhibit the activity of myogenic regulatory factors, thereby maintaining cells in an undifferentiated state and suppressing myogenic differentiation.

BMP signalling can be modified through endogenous secreted antagonists, including Noggin, which bind BMPs with a high affinity and interfere with BMP-BMPR interactions (Feng and Derynck, 2005). Research in murine MuSCs and myoblasts shows that exogenous BMP4 can sustain satellite cell division and prevent differentiation. Further, antagonising this pathway by exposing cultured myofibres to Dorsomorphin, an inhibitor of Smad1/5/8 phosphorylation, can induce premature differentiation (Friedrichs et al., 2011; Ono et al., 2011). These findings from murine models highlight the BMP pathway as a critical regulatory axis for controlling the balance between proliferation and differentiation during myogenesis and as a potential genetic target for fine tuning cell fate during a CM bioprocess. Overexpression of BMPs, such as BMP4, during proliferation of MuSCs may increase the maximum cell density by inhibiting MRF-mediated gene expression and maintaining the satellite cell pool (Ahmad et al., 2023).

Similarly to BMP signalling, Notch signalling maintains the satellite cell pool by repressing MyoD expression, primarily through the transcriptional repressor Hes1 (Gioftsidi et al., 2022). Notch signalling is an evolutionarily conserved pathway implicated in the regulation of myogenesis in vertebrate embryogenesis and *in vitro* cell culture models. Mechanistically, activation of Notch signalling involves the cleavage of its receptor by γ-secretase which releases Notch intracellular domain (NICD). The NICD translocates to the nucleus to activate downstream targets (Figure 2B). Activation of Notch signalling in C2C12s by treatment with the Notch ligand Delta1 can inhibit expression of MRFs, block myoblast fusion and prevent the formation of myotubes (Buas et al., 2009; Huot et al., 2020). Concordantly, mutations of Delta1 impair signalling and lead to a loss of myogenic progenitors and excessive myogenic differentiation (Schuster-Gossler et al., 2007; Wen et al., 2012). Further, treatment of C2C12s with a γ-secretase inhibitor (GSI) induced hypertrophy by increasing myonuclear accretion and myotube area in both differentiating and differentiated cells (Huot et al., 2020).

Similarly, GSI treatment increased protein synthesis in myotubes, likely mediated by activation of the anabolic Akt/mTOR pathway (see Section 4.2).

Conversely, and in the context of CM, Notch inhibition by a GSI has been shown to promote cell cycle exit and improve fusion index in bovine satellite cells (Melzener et al., 2024). Taken together, these findings highlight the potential of BMP and Notch signalling manipulation in fine tuning myogenic cell line proliferation and differentiation. Temporally modulating these pathways under inducible promoters may offer an effective strategy for simultaneously enhancing biomass accumulation during the proliferation phase and improving commitment to the myogenic lineage during differentiation, while removing the need for small molecule inhibitors in culture media. However, it must be noted that targeting these pathways through genetic engineering should be approached with caution. BMP signalling is known to have pleiotropic effects on cellular functions including apoptosis, migration, and extracellular matrix production (Friedrichs et al., 2011). Because these processes are interconnected, modifying BMP activity to enhance proliferative capacity may have unwanted off-target effects. Further, aberrant expression of BMPs or mutations in their associated signalling pathways have been described in various cancers and some BMPs have been linked with promoting the invasiveness of cancer cells (Dituri et al., 2019). It is important to consider the safety and public acceptance of edible cell lines generated through modification of pathways associated with cancer. This will be further discussed in Section 5.

## 3 Increasing differentiation efficiency

Once sufficient cell number has been achieved, efficient differentiation of MuSCs into mature myotubes will be important for generating the desired protein content for CM products. *In vitro*, MuSCs often exhibit incomplete or slow differentiation, with *in vitro* fusion indices of around 50% compared to almost complete differentiation in *in vivo* myogenesis (Melzener et al., 2024). Targeted genetic engineering can accelerate myotube formation, increase protein accumulation, and ensure more consistent product quality. To date, several patents have been filed in this space (Faram et al., 2021; Genovese et al., 2021). This section explores the key cell signalling pathways and genetic targets that have been leveraged to optimize the transition from proliferating myoblasts to fully differentiated muscle cells or deserve further consideration in this context.

### 3.1 The myogenic regulatory factors (MRFs)

Differentiation of skeletal muscle cells during embryogenesis and postnatal myogenesis is controlled by the myogenic regulatory factors (MRFs) Myf5, MyoD, myogenin and MRF4 (Braun and Gautel, 2011). The MRFs are members of the basic helix-loop-helix (bHLH) family of transcription factors that bind to cis-regulatory modules (CRMs) and regulate the expression of muscle-specific genes, including desmin, actin and myosin heavy chain genes. The temporal and spatial expression of MRFs forms the core of the transcriptional network that establishes the myogenic lineage and maintains the terminal myogenic phenotype. MuSCs are CD56^+^, PAX7+ and MyoD-cells responsible for muscle repair and regeneration in post-natal skeletal muscle (Soleimani et al., 2012; Olenic et al., 2024). Pax7 activates genes involved in the inhibition of differentiation and the regulation of proliferation, resulting in the maintenance of an adult MuSC phenotype which can activate upon muscle injury or during *in vitro* expansion to give rise to proliferating MyoD + myoblasts (Segalés et al., 2016). In the right conditions, these cells also express Myf5, MRF4 and myogenin to progress into the myogenic lineage and will eventually fuse their membranes to form multinucleated myotubes which can mature into myofibres (Figure 1).

The role of MRFs in cell cycle progression and differentiation has been studied most extensively in murine cell lines (Kassar-Duchossoy et al., 2005; Soleimani et al., 2012; Cai et al., 2020). In the context of CM, the early-stage MRF MyoD has been the primary transcription factor to be explored for the controlled induction of myogenesis, with inducible expression systems offering a means to temporally regulate differentiation. For instance, a recent patent was filed for a system controlling myogenic transcription factors, including MyoD, via optogenetics (Kent, 2024). In this system, light-controllable transcription activators or repressors allow for precise, temporal induction of myogenesis in suspension culture. Similarly, Biressi et al. (2023) filed a patent reporting the temperature-controlled expression of myogenic/adipogenic master regulators, namely MyoD and PPARy, in porcine pericytes (Biressi et al., 2023). Inducible systems offer a substantial advantage over constitutively activated or repressed genes for a CM bioprocess to allow for programmable, stage-specific optimisation of cell performance. Further, methods such as light- or temperature-inducible systems offer advantages over chemically inducible systems–such as doxycycline- or tetracycline-based promoters–which can bring cost, environmental and toxicity concerns, limiting their application in CM processes (Post et al., 2020).

While multiple MRFs orchestrate myogenic differentiation, MyoD has emerged as the most practical target for controlled induction in cultivated meat systems. Its pivotal role in committing MuSCs to the myogenic lineage, together with existing inducible systems, enables precise temporal regulation without compromising proliferation. Future strategies could include multiplexed control of MyoD alongside other MRFs or co-factors, tuneable synthetic promoters that enable precise control over the timing of MyoD expression for dynamic differentiation, and integration with metabolic or signalling pathway modifications to further optimize protein yield.

### 3.2 The MEK/ERK pathway

In adult skeletal muscle, periodic repair and replacement of muscle fibres by MuSCs is necessary to maintain functional integrity of the tissue. Growth factors, including insulin-like growth factors (IGFs) or FGFs, stimulate MuSC proliferation and differentiation into mature muscle fibres. IGF-1 and -2 interact with transmembrane IGF receptors, activating two major signalling cascades–the MEK/ERK pathway and the PI3K/AKT pathway (Figure 3). The PI3K/AKT pathway is the primary driver of protein anabolism in skeletal muscle and promotes differentiation and hypertrophy (an increase in cell size) (see Section 4.2), while early activation of the MEK/ERK pathway–a serine/threonine phosphorylation cascade initiated by the membrane-bound GTPase Ras–promotes proliferation and is important for hyperplastic growth (an increase in cell number). In the context of CM, overexpression of a constitutively active Ras mutant (Ras^G12V^) eliminated the need for exogenous FGF-2 by activating the MEK/ERK pathway (Stout et al., 2024).

**FIGURE 3:**
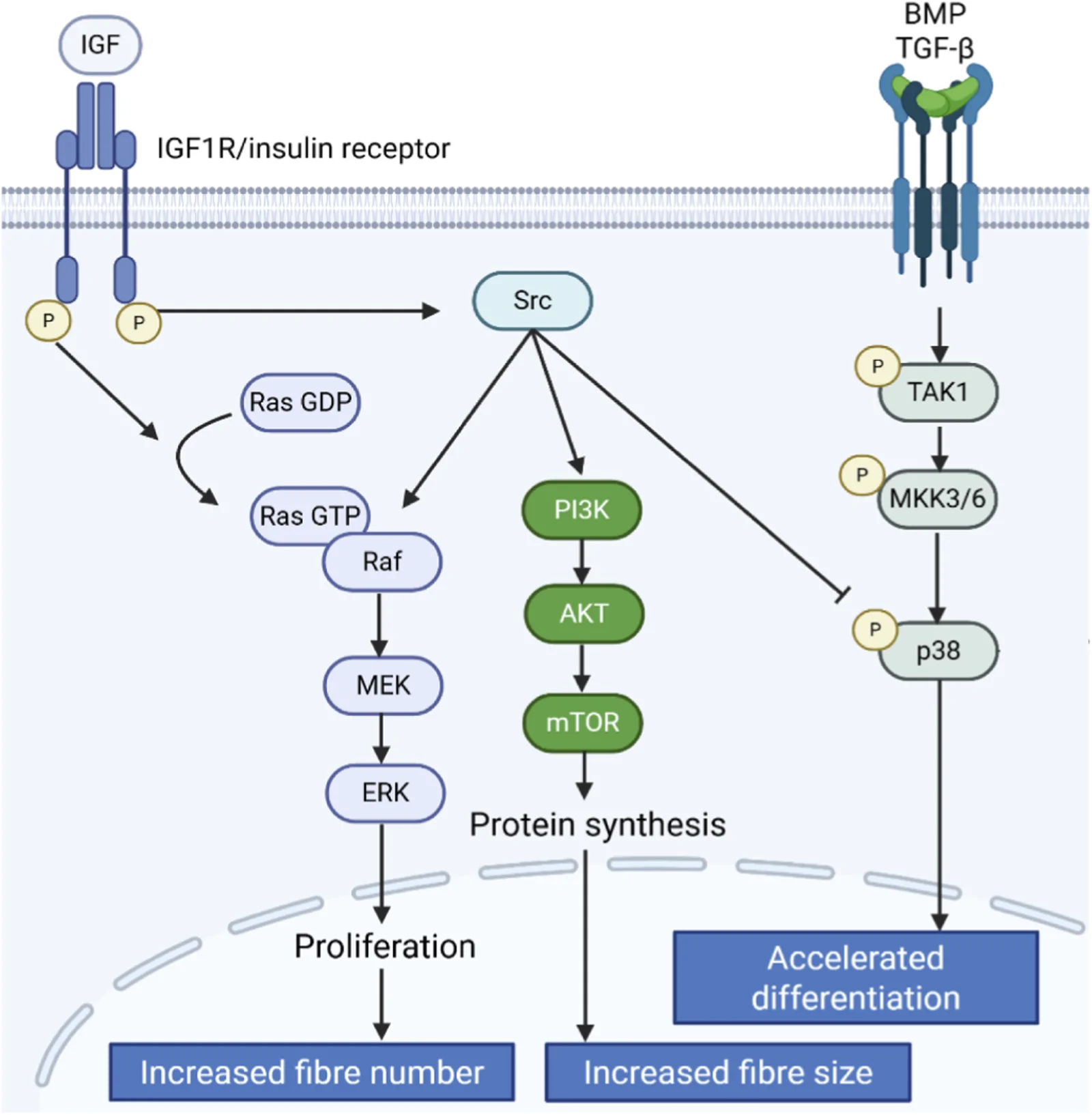
The MEK/ERK pathway drives cell proliferation. Src is an upstream activator of the MEK/ERK and PI3K/Akt signalling pathways and promotes cell survival and proliferation. The p38 MAPK pathway is activated by stress signals and promotes cell cycle arrest via inhibition of the MEK/ERK pathway and induces MyoD expression.

While the MEK/ERK pathway is important for reaching high cell densities, its inhibition is necessary for differentiation to occur. Ras and the upstream proto-oncogene c-Src–a non-receptor tyrosine kinase–act as pro-growth signals by pushing cells out of quiescence, maintaining them in a dividing, progenitor state (Boriack-Sjodin et al., 1998; Lu et al., 2016; Vatansever et al., 2020). Lim et al. (2007) demonstrated that inhibition of c-Src–by treatment with small molecule pharmacologic inhibitors or by expression of a dominant-negative c-Src–suppressed the MEK/ERK pathway while instead activating p38 MAPK, inducing differentiation even in proliferation medium (Lim et al., 2007). In the context of CM, pharmacological inhibition of p38 MAPK has been shown to delay differentiation and increase proliferative capacity of chicken MuSCs (Ryu et al., 2023). Further, Melzener et al. (2024) showed that MEK inhibition with a small molecule inhibitor reduced the number of bovine MuSCs that remained in an undifferentiated ‘reserve’ state and promoted cell cycle exit, increasing commitment to the myogenic lineage.

While inhibiting MEK remains a promising approach to increasing cell cycle exit during the differentiation phase of a CM bioprocess, the effects of this modification on cell viability and survival must also be evaluated.

## 4 Inducing hypertrophy *in vitro*

In addition to increasing total cell number or improving myogenic differentiation efficiency, inducing hypertrophy *in vitro* is a potential strategy for increasing protein yield and more closely mimicking skeletal muscle in a CM bioprocess. Once sufficient cell numbers are reached and differentiation is induced, myoblasts fuse into myotubes. This increases the total biomass per cell and more closely aligns the protein composition with that of mature muscle tissue, compared to undifferentiated cells (Guan et al., 2023). Muscle hypertrophy is the enlargement of existing myotubes or fibres driven by a net protein synthesis, which is regulated by a combination of growth factor signalling, cytokines, and transcriptional pathways.

This section will explore these regulatory networks–including canonical and non-canonical Wnt signalling, the PI3K/Akt/mTOR axis, and myokine-mediated regulation–in the context of their roles in promoting myotube enlargement and skeletal muscle protein synthesis. Although myotube enlargement may represent a method of increasing protein yield from a CM bioprocess, it must also be noted that larger myotubes may have altered metabolism, consume more oxygen and nutrients or create diffusion limitations. Increasing the protein content per cell may not necessarily increase the total protein yield or do so in a cost effect manner. These considerations should be further explored in the context of the proposed genetic alterations below.

### 4.1 Wnt signalling

Wnt is a family of signalling molecules involved in embryogenesis and adult tissue repair by activating canonical and non-canonical Wnt signalling cascades in target cells. Multiple studies have described the induction of muscle hypertrophy, characterised by an increase in myofibre size and skeletal muscle mass, by activation of canonical Wnt signalling (Brack et al., 2007; Han et al., 2011).

In canonical Wnt signalling, Wnt ligands bind to their respective Frizzled (Fzd) receptors, which activates Dishevelled (Dsh) and heterotrimeric G proteins. This inactivates the destruction complex–consisting of Axin, glycogen synthase kinase 3β (GSK-3β) and Adenomatous polyposis coli (APC) – which would otherwise target β-catenin, a co-activator necessary for myogenic differentiation, for degradation (Katoh and Katoh, 2007; Nusse, 2008; Sethi and Vidal-Puig, 2010; Clevers and Nusse, 2012). This results in accumulation of cytoplasmic β-catenin which subsequently translocates to the nucleus where it binds members of the LEF and TCF transcription factors to induce context-dependent Wnt/β-catenin target genes (Abu-Elmagd et al., 2010).

Several studies have implicated GSK-3β as a negative regulator of skeletal muscle hypertrophy (Vyas et al., 2002; Van Der Velden et al., 2007). Rochat et al. (2004) showed that inhibition of GSK-3β by small molecule inhibitors or LiCl results in accumulation of nuclear β-catenin and induces myotube hypertrophy, enhancing insulin-induced differentiation of C2 myogenic cells and human myoblasts. When reserve cells are cooperatively activated by GSK-3β inhibition and insulin, they are directly recruited into preexisting myotubes, suggesting insulin and Wnt/β-catenin pathways cooperate during myogenesis (Figure 4, pink) (Rochat et al., 2004). Recent work suggests that inhibition of GSK-3β with small molecule inhibitors improves proliferation and differentiation of C2C12s (Jiang et al., 2025). Genetic engineering of canonical Wnt signalling, for instance by targeted knockdown of GSK-3β, may represent a more desirable method than media supplementation with small molecule inhibitors by reducing variability, complexity and cost of culture media while avoiding potential concerns associated with small molecule or chemical residues.

**FIGURE 4:**
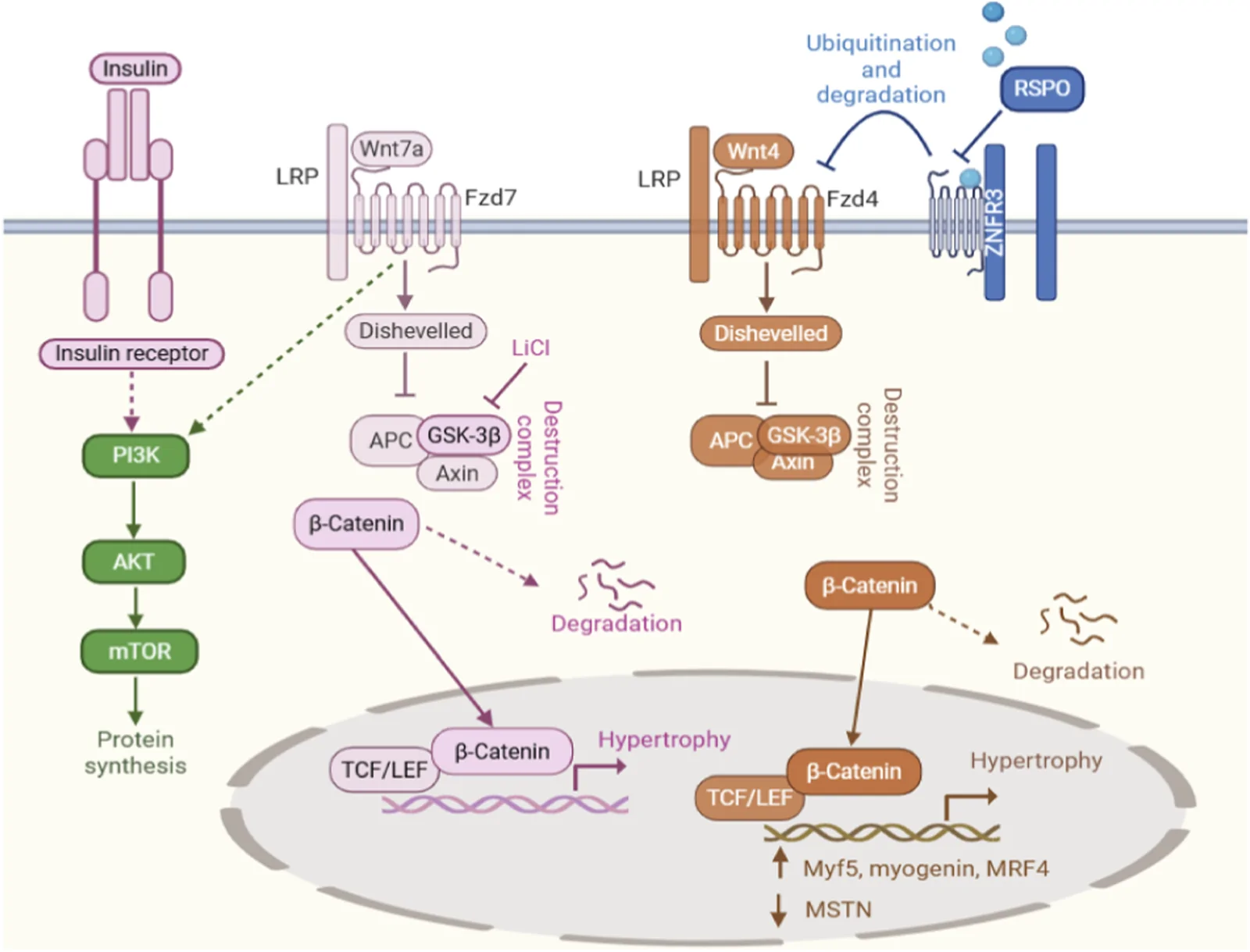
Wnt signalling in myogenesis and muscle hypertrophy. During muscle regeneration, canonical Wnt signalling induces hypertrophy which can cooperate with insulin-dependent signalling (pink). R-spondins can promote canonical Wnt signalling by preventing degradation of Fzd (blue). Wnt7a can also act via non-canonical signalling, activating the PI3K/AKT pathway independently of β-catenin (green). Canonical signalling by Wnt4 can promote hypertrophy by increasing expression of myogenic regulatory factors (MRFs) and negatively regulating expression of the myogenic inhibitor myostatin (MSTN) (orange).

Wnt signalling is amplified by a group of secreted proteins known as R-spondins (RSPOs) which prevent the degradation of Wnt receptors, exponentially boosting cell sensitivity to Wnt ligands (Lebensohn and Rohatgi, 2018). Further, RSPO2 and RSPO3 have been shown to promote myotube formation *in vitro* (Han et al., 2011). Upregulation of RSPOs, in combination with overexpression of Wnt ligands, may represent a genetic target for improving myogenic efficiency (Figure 4, blue).

By contrast to canonical signalling, the less well characterised non-canonical Wnt signalling does not require the transcriptional activity of β-catenin, and include the PCP, Wnt/Ca^2+^ and the PI3K/AKT pathway (Figure 4, green). Von Maltzhan et al. (2012) implicated Wnt7a ectopic expression or application in inducing skeletal muscle hypertrophy via non-canonical Wnt signalling, independent of β-catenin, by binding to Fzd7 and increasing phosphorylation of PI3K, activating the downstream AKT/mammalian target of rapamycin (mTOR) anabolic pathway independent of IGF receptor activity (Von Maltzahn et al., 2012). The authors hypothesised that this was due to the presence of a myotube specific receptor complex containing Fzd7 and PI3K–not present in proliferating myoblasts–which mediates the activation of AKT.

Wnt4 is also thought to act primarily via non-canonical signalling, however Steelman et al. (2006) demonstrated that*, in vitro*, Wnt4 induces hypertrophy through canonical Wnt signalling, marked by increased expression of the myogenic regulatory factors Myf5, myogenin and MRF4 (Figure 4, orange). Overexpression of Wnt4 in chicken embryos improved differentiation both by activating the canonical Wnt/β-catenin pathway and by negatively regulating myostatin (MSTN) expression (see Section 4.3) (Steelman et al., 2006). In the context of CM, Wnt7a is particularly attractive because it activates hypertrophy independently of IGF signalling, potentially reducing reliance on costly recombinant growth factors. However, whether this advantage is retained in agriculturally relevant cell types remains unknown.

Harnessing hypertrophic signalling offers significant potential to improve the protein content and functional properties of cultured muscle. However, although activation of canonical Wnt signalling promotes hypertrophy, sustained pathway activation is known to also impact proliferation, stem cell maintenance and fibrogenesis in a tissue- or cell-type dependent manner (Habib and Acebrón, 2022). Therefore, constitutive activation may generate undesirable phenotypes or impair long-term cell stability. Engineering strategies should therefore prioritise transient or stage-specific modulation rather than permanent pathway activation.

### 4.2 The PI3K/AKT/mTOR pathway

The PI3K/AKT/mTOR pathway is a key signalling cascade in promoting differentiation and activation of this pathway via IGF-1 is widely considered the primary route to skeletal muscle hypertrophy (Morissette et al., 2009). Following growth factor binding to its receptor tyrosine kinase (RTK), the activated RTK recruits and activates PI3K which phosphorylates PIP2 to PIP3 at the cell surface membrane. PIP3 serves as a docking site for AKT which is phosphorylated by phosphatidylinositol-dependent protein kinase 1 (PDK1). In turn, AKT phosphorylates several substrates responsible for growth processes including protein synthesis, proliferation and differentiation. AKT functions as a regulator of protein synthesis by activating the protein kinase mTOR and by inhibiting forkhead box O (FoxO) transcription factors involved in protein degradation (Crossland et al., 2008). PI3K signalling is negatively regulated by PTEN, which dephosphorylates PIP3 to PIP2, preventing AKT activation (Song et al., 2012).

Activation of PI3K/AKT/mTOR downstream of IGF-1 has been shown to induce hypertrophy in the model cell line C2C12, as well as *in vivo* (Yeh et al., 2014). Overexpression of IGF-1 in bovine MuSCs has been shown to increase myogenic gene expression without the need for exogenous IGF-1 supplementation (Li et al., 2024). In addition to anabolic growth factors, *in vivo* hypertrophic growth is also regulated by the secretion of cytokines, such as interleukin-6 (IL-6), by macrophages which are recruited to the damaged region of muscle following exercise-induced inflammation (Hawke and Garry, 2004). Studies show that with an enhanced macrophage response, there is an increase in satellite cell proliferation and differentiation (see Section 4.3) (Lescaudron et al., 1999; Nathan, 2012). While the use of macrophages for co-culture is beyond the scope of this review, an understanding of this mechanism can be used to inform target selection for cell line engineering.

Recent work into plant secondary metabolites as culture media supplements identified the flavonoid luteolin as pro-myogenic, as indicated by improved fusion index of porcine myoblasts (Guan et al., 2023). Pharmacological inhibition of PI3K activity attenuated this effect, suggesting luteolin increases activity of the PI3K/AKT/mTOR pathway. Although the addition of plant-derived compounds to culture media represents a promising avenue for inducing hypertrophy, several challenges exist, including variability and production costs (Wali et al., 2024). Enhancing PI3K/AKT signalling via cell line engineering, for instance through inhibition of the negative regulator PTEN, represents an attractive strategy for enhancing protein content in CM products. However, knockout of PTEN has previously been shown to enhance proliferation but prevent differentiation in bovine mesenchymal cells (MSCs) (Zirman et al., 2025), so more research is needed to optimise the temporal disruption of this gene in different cell types. Further, because PTEN functions as a major tumour suppressor gene, permanent genetic disruption may raise concerns regarding genomic stability, regulatory approval and consumer acceptance. Partial suppression or inducible regulation may therefore prove more suitable than complete knockout and genetic testing should be a priority to ensure cell line stability.

### 4.3 Myostatin and other myokines

Myokines are cytokines with anabolic potential that play important roles in myogenesis, including IL-4, IL-6 and leukaemia inhibitory factor (LIF) (Shaikh et al., 2021). Myostatin (MSTN) is a myokine belonging to the TGF-β family that negatively regulates myogenesis and is well conserved across species (Shaikh et al., 2021). MSTN acts by binding activin receptor type IIA and IIB receptors, inducing phosphorylation of Smad3. Activated Smad3 forms a complex with Smad4 which is translocated into the nucleus where it blocks MyoD from binding to E-box elements in muscle-specific genes (Langley et al., 2002; Eom et al., 2025). MSTN arrests muscle cells in the G1 and G2 phase of the cell cycle via the upregulation of p21 which inhibits cell proliferation. This is, in part, mediated through the p38 MAPK pathway, however MSTN has also been shown to directly inhibit muscle differentiation by specifically inducing Smad3 phosphorylation which downregulates MyoD expression (see Section 2.2) (Langley et al., 2002; Chang, 2007). Consistent with the inhibitory role of MSTN in myogenesis, exogenous supplementation with the plant-derived flavonoid quercetin enhanced myogenic differentiation in bovine, porcine and chicken MuSCs by inhibiting the MSTN/SMAD2/3 signalling pathway (Ahmad et al., 2024).

Naturally occurring mutations in MSTN, leading to non-functional proteins, have been identified in domestic animals, including pigs, cows and sheep and are associated with the “double-muscling” phenotype. Several groups have generated MSTN knockout (KO) cattle, improving muscle phenotype and Eom et al. (2025) were the first to show that primary cells from MSTN KO cows exhibited enhanced myogenic potential *in vitro*. In concordance, the authors found that treatment of immortalised fibroblasts with MSTN reduced MyoD-induced myogenesis, while MSTN KO increased it. However, it is important to note that these results were obtained from non-myogenic cells which were modified to be myogenic and the effect of MSTN KO on myogenic lineages remains to be elucidated. Further, it is unclear whether hypertrophy induced by constitutive MSTN KO may be accompanied by altered metabolic demand or impaired proliferation that could reduce manufacturing efficiency. These trade-offs have yet to be systematically evaluated.

LIF is part of the IL-6 family of cytokines and is produced when *in vitro* myotubes are electrically stimulated (Broholm et al., 2008). *In vivo* studies have implicated LIF as a stimulator of satellite cell proliferation and a contributor to hypertrophy. In LIF knockout mice, the hypertrophic response to functional overload was stunted, and recovered upon restoration of physiological concentrations of LIF (Spangenburg and Booth, 2002). Further, acute administration of IL-6 and LIF in *in vitro* cultures of differentiated C2C12 myotubes induced protein synthesis likely via the activation of the PI3K/AKT pathway and signal transducer and activator of transcription 3 (STAT3). Active STAT3 induces expression of suppressor of cytokine signalling 3 (SOCS3), a key negative feedback regulator of cytokine signalling (Figure 5). Knockdown of SOCS3 increased both cytokine-induced and basal protein synthesis, suggesting IL-6 or LIF-induced SOCS3 negatively regulates protein synthesis and highlighting SOCS3 as a potential genetic target for improving protein production in CM cell lines (Gao et al., 2017).

**FIGURE 5:**
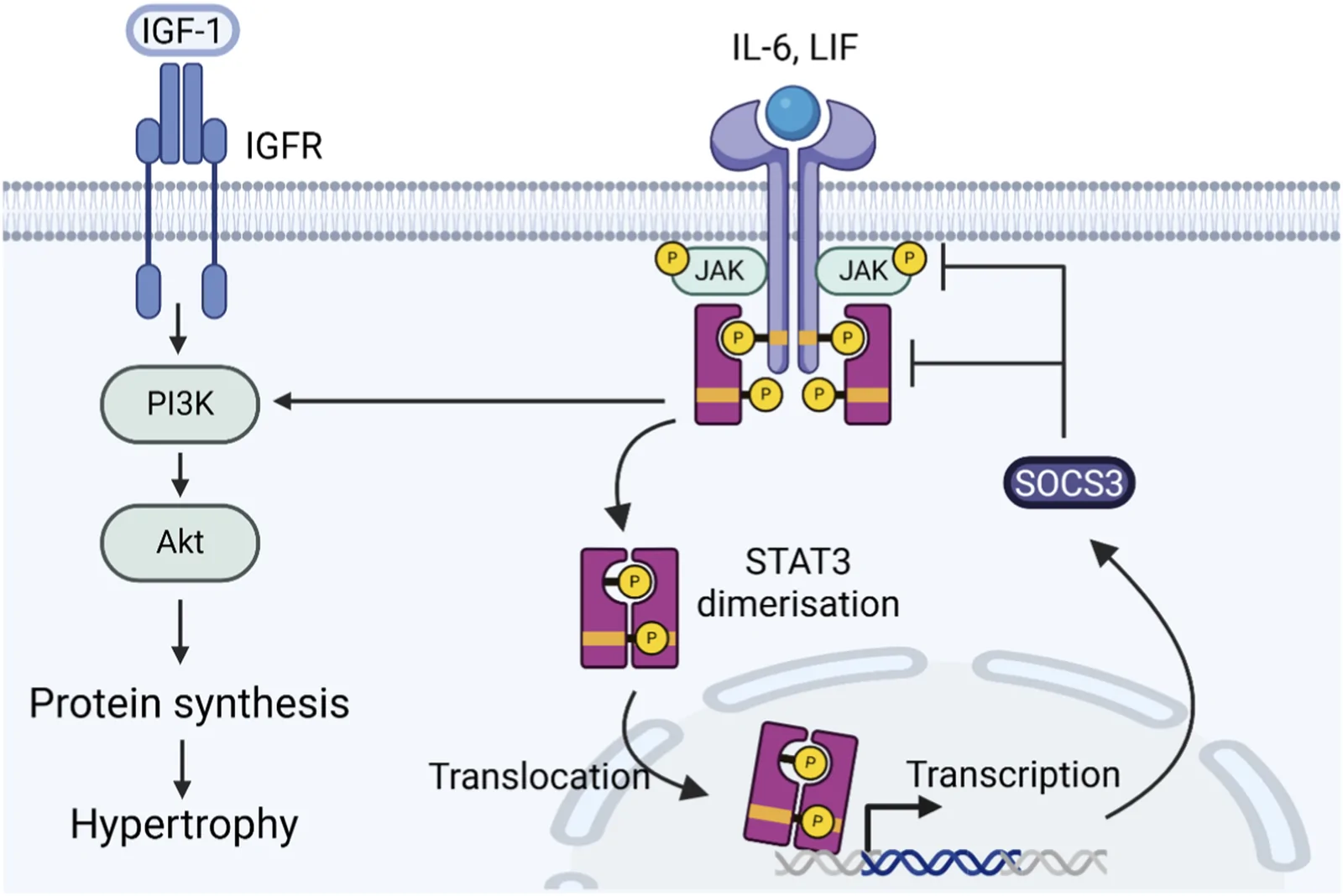
The roles of myokines in skeletal muscle protein synthesis and hypertrophy. In response to resistance exercise, macrophages infiltrate damaged muscle and release cytokines like IL-6, promoting satellite cell proliferation and muscle hypertrophy alongside IGF-1 signalling. *In vitro*, myokines such as LIF and IL-6 enhance myogenic differentiation and protein synthesis through the AKT/mTOR pathway.

Myokine signalling represents a promising and relatively underexplored avenue for CM cell line engineering. However, while inhibition of negative regulators such as MSTN provide a promising strategy to enhance differentiation and hypertrophy, its effects are highly context-dependent and may introduce trade-offs between hypertrophy, proliferative capacity and metabolic demand. Further, the transient nature of cytokine responses and the presence of negative feedback mechanisms, such as SOCS3, highlight the need for precise temporal control. Further investigation in edible cell lines is needed to determine whether these pathways can improve cultivated meat production efficiency without compromising overall cell performance.

Table 1 summarises the key genetic targets that may be considered for enhancing myogenesis in muscle-derived cell lines as discussed above. Although these targets have either been promising in early CM research or show biological relevance, not all are equally attractive engineering targets. Many of the identified genetic targets have been extrapolated from model systems, such as C2C12s. It is important to note that species-specific differences in signalling responses and differentiation capacity, as well as cell type-specific differences such as immortalisation phenotype could substantially limit the translational relevance of the discussed targets.

**TABLE 1:** Key regulatory pathways and genetic engineering targets for improving myogenesis.

Regulatory pathway	Target	Intended phase	Evidence informing intervention	Species/cell type	Evidence in CM-relevant cells	Translational considerations	References
BMP/Smad	↑BMP4	Proliferation	Pharmacological	Murine myoblast	N	Evidence primarily from non-CM model systems; effects on proliferation/differentiation may be context dependent	Katagiri et al., 2002; Ono et al., 2011
MEK/ERK	↑Ras	Proliferation	Genetic	Bovine MuSCs	Y	Evidence in agriculturally relevant cells; promotes proliferation, but constitutive Ras activation raises concerns regarding pleiotropic effects and genomic/safety considerations	Stout et al., 2024
	↓p38 MAPK	Proliferation	Pharmacological	Chicken MuSCs	Y	Evidence in agriculturally relevant cells; inhibition may extend the proliferative phase, but pharmacological evidence does not establish suitability for constitutive genetic suppression. Temporal modulation may be preferable to avoid impairing differentiation	Ryu et al., 2023
Notch	↑Delta1	Proliferation	Pharmacological	C2C12s	N	Well-established role in maintaining progenitor state, but evidence is largely from model systems; prolonged overexpression may interfere with subsequent differentiation	Buas et al., 2009; Wen et al., 2012)
	↓γ-secretase	Differentiation/hypertrophy	Pharmacological	C2C12s	N	Inhibition can promote hypertrophy, but potential for substantial pleiotropic effects; temporal modulation may be preferable	Huot et al., 2020
MEK/ERK	↓MEK	Differentiation	Pharmacological	Bovine MuSCs	Y	Evidence in bovine cells; transient inhibition of MEK can promote differentiation and cell-cycle exit; modulation can be temporally restricted to the differentiation phase	Michailovici et al., 2014; Ding et al., 2018; Eigler et al., 2021; Melzener et al., 2024
	↑p38 MAPK	Differentiation	Pharmacological	C2C12s	N	Potential to induce differentiation even in proliferation medium; no CM-relevant evidence	Lim et al., 2007
MRFs	↑MyoD	Differentiation	Genetic	Porcine pericytes; pluripotent/mesenchymal stem cells proposed; fibroblasts	Y*	Strong master-regulator effect, but constitutive expression may cause premature differentiation and compromise expansion; inducible/temporal expression may be preferable	Biressi et al., 2023; Kent, 2024
Wnt signalling	↑Wnt ligands e.g., Wnt7a	Differentiation/hypertrophy	Genetic/pharmacological	C2C12s and *in vivo* mouse model	N	Potential to promote differentiation and hypertrophy, but broad effects on cell fate and tissue homeostasis reduce predictability; limited CM-relevant evidence	Rochat et al., 2004; Han et al., 2011; Von Maltzahn et al., 2012
PI3K/AKT/mTOR	↑IGF1	Differentiation/hypertrophy	Genetic	Bovine MuSCs	Y	Evidence in agriculturally relevant cells and direct relevance to protein synthesis/hypertrophy	Li et al., 2024
	↓PTEN	Differentiation/hypertrophy	Genetic	*In vivo* mouse model	N	Potentially effective but constitutive disruption of a negative regulator may produce broader signalling effects and raises additional genomic/safety considerations	Yue et al., 2016
Myokine signalling	↓ MSTN	Differentiation/hypertrophy	Pharmacological and genetic	C2C12s; bovine, porcine, and chicken MuSCs	Y	Strong relevance to muscle hypertrophy and existing CM evidence; however, effects may include trade-offs between hypertrophy, proliferation and metabolic demand; long-term effects require evaluation	Langley et al., 2002; Ahmad et al., 2024; Eom et al., 2025
	↓ SOCS3	Differentiation/hypertrophy	Genetic	C2C12s	N	Potential to enhance cytokine-induced and basal protein synthesis, but evidence is limited to C2C12s; the broader effects of sustained SOCS3 suppression and the need for temporal control require further investigation in CM-relevant cells	Gao et al., 2017

* CM-relevant patent evidence; experimental validation was performed in porcine pericytes and fibroblasts, while broader application to pluripotent/mesenchymal stem cells is described in the patents.

The arrows represent overexpression (↑) or silencing (↓) of the suggested target to achieve the desired phenotype. The intended phase refers to either proliferation, differentiation or hypertrophy. Two different types of evidence are highlighted–either genetic or pharmacological, referring to existing evidence based on genetic modifications or exogenous application of small molecules or ligands. Whether there is evidence from CM-relevant cell types (Yes; Y, No; N) and the translational considerations of each intervention are also listed.

To assess the translational potential of the reviewed genetic targets for CM, we consider several factors: the strength of evidence in agriculturally relevant cell types, the potential for temporal or reversible modulation, and potential implications for genomic stability. The translational potential of a genetic target is distinguished from commercial or regulatory readiness, as the latter is evolving and cannot currently be predicted reliably from the available evidence. Overall, many of the targets identified require further validation in agriculturally relevant cell lines and assessment of their compatibility with scalable CM bioprocesses. In particular, manipulation of pleiotropic pathways such as Wnt or TGF-β is likely to require greater optimisation because of their broader effects on cell fate and tissue homeostasis.

## 5 Regulatory and ethical considerations of cell line engineering for cultivated meat

Although the pathways discussed in this review represent promising targets for improving myogenesis based on research in parallel fields–such as regenerative medicine and tissue engineering–or in early-stage CM research, several critical issues must be considered to enable translation to CM production.

Firstly, the use of genetic engineering itself as a method for cell line development. Genetic engineering can include a variety of manipulations ranging from transgenic insertions of genes from unrelated organisms to the precise edits of single base pairs by CRISPR-based systems (Riquelme-Guzmán et al., 2024). Next-generation gene editing tools emerging from the gene therapy space, such as CRISPR systems have opened the door to precise genome rewriting in the absence of double-strand breaks, reducing off-target effect (Naeem et al., 2020). Such precise editing results in cell lines that are often indistinguishable from cell lines generated through random mutagenesis or selective breeding. While precise gene editing may offer advantages over transgene insertion, it remains to be seen whether regulatory or consumer bodies will more readily accept products developed by such techniques.

Secondly, the public perception around the safety of gene edited or genetically modified foods. Although genetic engineering has already been applied in livestock species (Park, 2023), its application in CM has raised concerns about the safety of consuming cell lines. These concerns are primarily associated with immortalised cell lines due to the overlap between the gene modifications involved in the immortalisation process and those that are dysregulated in cancer. Some genes discussed in this review, including Ras and PTEN, are also dysregulated in cancer and, therefore, may not be suitable targets for this application. Despite this perceived fear, it is important to state that cancer cells are described according to the acquisition of multiple hallmarks including genome instability, resisting cell death and sustained angiogenesis, not only the ability to surpass the Hayflick limit which may be presented by immortal cell lines for CM (Hanahan and Weinberg, 2011). Thus, there is a fundamental difference between cancer cells and the highly proliferative yet phenotypically stable and tightly controlled cells that are required for CM production. It is also important to note that there is no evidence of dietary DNA being incorporated into consumer DNA nor cancer being transmitted via ingestion of cancerous tissue from conventional meat.

Thirdly, the off-target genomic alterations and the impact on genome stability over extended passages of a given genetic alteration must be considered for a CM bioprocess. Although some genetic or phenotypic drift is expected during long-term expansion of cell lines, some genetic alterations may be associated with an increased risk of further mutation and excessive genomic instability may compromise manufacturing reliability. Unintended genomic alterations may alter cellular metabolism, interfere with protein expression, and produce potentially harmful metabolites. The use of temporal modulation, such as optogenetics or other inducible promoters, may represent a valuable tool to reach a desired phenotype with few off-target effects. Further, the use of well-maintained master cell banks and working cell banks will ensure consistency and traceability across production runs (Mia et al., 2026). A recent review drawing on publicly available dossiers from CM companies revealed large variability in testing strategies for genomic stability and tumorigenicity evaluation (Mia et al., 2026). It will still be important for regulatory bodies to ensure CM companies align on their assessment criteria and that any hazards associated with changing cell phenotype or genome instability are identified and closely regulated.

To date, several CM companies–including Meatable and SciFi Foods–have publicized the benefits of cell line engineering for the generation of high performing cell lines with improved differentiation. UPSIDE Foods’ first-generation chicken fillet product successfully completed the FDA’s pre-market safety consultation, which included evaluation of the company’s cultured cell material. Following jurisdiction-specific standards and regulations, other countries may also consider engineering approaches for the development of CM.

## 6 Conclusions and outlook

Decoupling meat production from conventional animal agriculture has the potential to satisfy an increasing demand for protein while mitigating the negative effects on planetary and public health associated with farming animals at an industrial scale. The use of genetic engineering to fine tune myogenesis represents a promising avenue for sustainable, low-cost scale up of CM technology enabling higher biomass and protein yield while ensuring bioprocess simplicity and consistency. These potential outcomes warrant further investigation across relevant cell types and bioprocesses.

This review has highlighted existing and emerging genetic targets for the development of high-performance muscle derived cell lines, including transcriptional regulators, intracellular signalling pathways and myokines, with the aim to improve temporal control and efficacy of myogenesis. It is important to note that no single genetic modification or pathway manipulation is likely to optimise myogenic cell line performance alone. Many of the pathways discussed interact extensively and dynamic engineering is likely to be more useful than constitutive overexpression or inhibition of genes, which may result in off target effects or genomic instability.

It must be acknowledged that for engineered cell lines to reach market, all stakeholders, including consumers and regulatory bodies, must be considered, and effective outreach and communication strategies will be required to achieve this goal. Moving forward, a transdisciplinary approach combining bioengineering and cell biology with social science and public engagement will be required to unlock the full potential of genetic engineering in making CM a viable alternative protein source.
